# Data Integrity–Based Methodology and Checklist for Identifying Implementation Risks of Physiological Sensing in Mobile Health Projects: Quantitative and Qualitative Analysis

**DOI:** 10.2196/11896

**Published:** 2018-12-14

**Authors:** Jia Zhang, Laura Tüshaus, Néstor Nuño Martínez, Monica Moreo, Hector Verastegui, Stella M Hartinger, Daniel Mäusezahl, Walter Karlen

**Affiliations:** 1 Mobile Health Systems Lab, Institute of Robotics and Intelligent Systems Department of Health Sciences and Technology ETH Zurich Zurich Switzerland; 2 Department of Epidemiology & Public Health Swiss Tropical and Public Health Institute Basel Switzerland; 3 University of Basel Basel Switzerland; 4 Universidad Peruana Cayetano Heredia Lima Peru

**Keywords:** physiological monitoring, data completeness, data quality, signal quality, medical sensors, implementation research, content analysis, mHealth, digital health

## Abstract

**Background:**

Mobile health (mHealth) technologies have the potential to bring health care closer to people with otherwise limited access to adequate health care. However, physiological monitoring using mobile medical sensors is not yet widely used as adding biomedical sensors to mHealth projects inherently introduces new challenges. Thus far, no methodology exists to systematically evaluate these implementation challenges and identify the related risks.

**Objective:**

This study aimed to facilitate the implementation of mHealth initiatives with mobile physiological sensing in constrained health systems by developing a methodology to systematically evaluate potential challenges and implementation risks.

**Methods:**

We performed a quantitative analysis of physiological data obtained from a randomized household intervention trial that implemented sensor-based mHealth tools (pulse oximetry combined with a respiratory rate assessment app) to monitor health outcomes of 317 children (aged 6-36 months) that were visited weekly by 1 of 9 field workers in a rural Peruvian setting. The analysis focused on data integrity such as data completeness and signal quality. In addition, we performed a qualitative analysis of pretrial usability and semistructured posttrial interviews with a subset of app users (7 field workers and 7 health care center staff members) focusing on data integrity and reasons for loss thereof. Common themes were identified using a content analysis approach. Risk factors of each theme were detailed and then generalized and expanded into a checklist by reviewing 8 mHealth projects from the literature. An expert panel evaluated the checklist during 2 iterations until agreement between the 5 experts was achieved.

**Results:**

Pulse oximetry signals were recorded in 78.36% (12,098/15,439) of subject visits where tablets were used. Signal quality decreased for 1 and increased for 7 field workers over time (1 excluded). Usability issues were addressed and the workflow was improved. Users considered the app easy and logical to use. In the qualitative analysis, we constructed a thematic map with the causes of low data integrity. We sorted them into 5 main challenge categories: environment, technology, user skills, user motivation, and subject engagement. The obtained categories were translated into detailed risk factors and presented in the form of an actionable checklist to evaluate possible implementation risks. By visually inspecting the checklist, open issues and sources for potential risks can be easily identified.

**Conclusions:**

We developed a data integrity–based methodology to assess the potential challenges and risks of sensor-based mHealth projects. Aiming at improving data integrity, implementers can focus on the evaluation of environment, technology, user skills, user motivation, and subject engagement challenges. We provide a checklist to assist mHealth implementers with a structured evaluation protocol when planning and preparing projects.

## Introduction

### Background

Limited access to adequate health care is a major burden in low- and middle-income countries and affects the poor most [1]. Centralized and outreach health care facilities are often sparsely available, difficult to reach, and overloaded. In addition, access to the health care centers can be costly, as patients often have to pay for transportation and compensate for the loss of income because of their absence from work [2]. Mobile health (mHealth) is a promising field that seeks to bring health care closer to the patient, thereby improving access and reducing costs because of its potential for a system-wide application [3]. We interpret mHealth as the use of mobile, digital communication technologies (eg, mobile phones) in medical and public health applications for effectively delivering health care and medical information [4]. Biomedical sensing using connected mobile sensors is an important but largely unexplored application in mHealth. It provides objective measurement of physiological parameters and facilitates more reliable diagnoses and assessments of patients. Physiological parameters that can currently be measured with mobile tools include blood pressure [5], respiratory rate (RR) [6], heart rate (HR) and electrocardiogram [7], peripheral capillary oxygen saturation (SpO_2_) [8], and blood glucose levels [9].

The integration of additional medical sensors into mHealth projects increases the technological complexity. Furthermore, users require additional skills and medical knowledge, whereas systems need to be purchased and maintained. Thus, these additional challenges need to be considered during the implementation of physiological monitoring projects. The validated use of medical sensors depends on well-defined working conditions and the adherence to standards to ensure correct sensor function and data quality. Sensor failures and motion artifacts are possible intermittent issues encountered and, therefore, when operating in remote settings, a basic understanding of medical sensing mechanisms is required for safe application of sensors and to identify faulty or noisy data at the point of use. It can be challenging to address these issues when inexperienced community health care workers with little or no prior knowledge about interpreting physiological signals are operating the sensors. Numerous mHealth projects have implemented physiological sensors, for example, pulse oximeters, for measuring SpO_2_ and HR, but none of them directly focused on evaluating the challenges associated with their implementation. Challenges were observed in clinical settings, that is, Hudson et al identified that the lack of training and nonfamiliarity with clinical alarms are barriers to apply pulse oximeters [10]. Furthermore, Spence et al identified different priorities across stakeholders [11], and English et al identified significant differences in observed errors between clinicians and nursing staff [12]. In summary, no research study has systematically examined the challenges of implementing physiological sensing and monitoring with mHealth tools.

As a consequence, no established methodology exists that would enable mHealth implementers to formally evaluate their projects and prevent implementation pitfalls with respect to physiological monitoring in low-resource settings. Although King et al organized focus group discussions with trained health care providers to identify challenges when managing pediatric pneumonia with pulse oximetry [13], their findings are country specific and limited to pulse oximeters. Wallis et al organized group discussions and proposed a roadmap for overcoming barriers of implementing image-based mHealth implementations [14], but their strategies are limited to image-based applications. On the other hand, Aranda-Jan et al applied the strengths, weaknesses, opportunities, and threats analysis method to review mHealth projects [15]. In addition, Eckman et al provided a conceptual strategy that involved all stakeholders into the design phase to assess the common failures of mHealth implementation [16]. However, both approaches did not explicitly address the challenges of physiological sensing and the specific risks associated to adding medical sensors to mHealth projects. The absence of a methodology or guideline during implementation could easily lead to overlooking domain-specific issues, evaluation errors, and the underestimation of risks and, therefore, prevent projects from achieving their goal of improving health outcomes.

We consider data integrity as the most important criterion for evaluating the risks of an mHealth project. Data integrity represents the faithfulness of information comprising criteria such as completeness, accuracy, relevance, consistency, usability, and reliability [17]. During unsupervised data collection, as it is frequently the case in mHealth, data completeness and consistency are critical quality metrics. Incomplete, poor, and missing data not only reduce the sample size but may also introduce bias or false conclusions. In clinical decision making, the signal quality and its reliability during physiological data collection using medical sensors are the most important factors [18]. Usability of a medical device is another component of data integrity that is associated with correct use and usage errors. International standards specify usability evaluation processes to reduce the risk of usability failures [19]. Poor usability can lead to the misuse of a medical device or a reduction of user engagement, resulting in unusable or missing data.

Due to the decentralized nature of mHealth, the assurance of data integrity is challenging [20]. High measurement uncertainty because of the lack of a controlled environment, unknown training status of the user, and higher risks for misuse of the technology require special attention. Although the goal of any mHealth implementation is to provide access to health services and, consequently, improve health outcomes, obtaining good data integrity with the provided technology is essential to positively influence these outcomes. Therefore, evaluating data integrity should not only be part of the evaluation of implementation success at the end of an mHealth study but considered and assessed already early in the preparation phase. Consequently, data integrity could serve as the central theme when framing a methodology for evaluating implementation challenges.

### Objectives

Our goal was to develop a methodology to systematically evaluate general risks and challenges of sensor-based physiological monitoring in mHealth and to avoid pitfalls before and during its implementation. Our specific aims in developing such a methodology were to (1) identify sources of low data integrity with a special focus on implementations that occur in remote or low-resource settings, (2) derive generalized risk factors that could guide a pre-implementation evaluation, and (3) provide an actionable tool to conduct such evaluation. The results could support implementers in evaluating their projects with regard to hidden risks and facilitate quality control early in the design and implementation of advanced mHealth tools.

## Methods

### Overview

**Figure 1:**
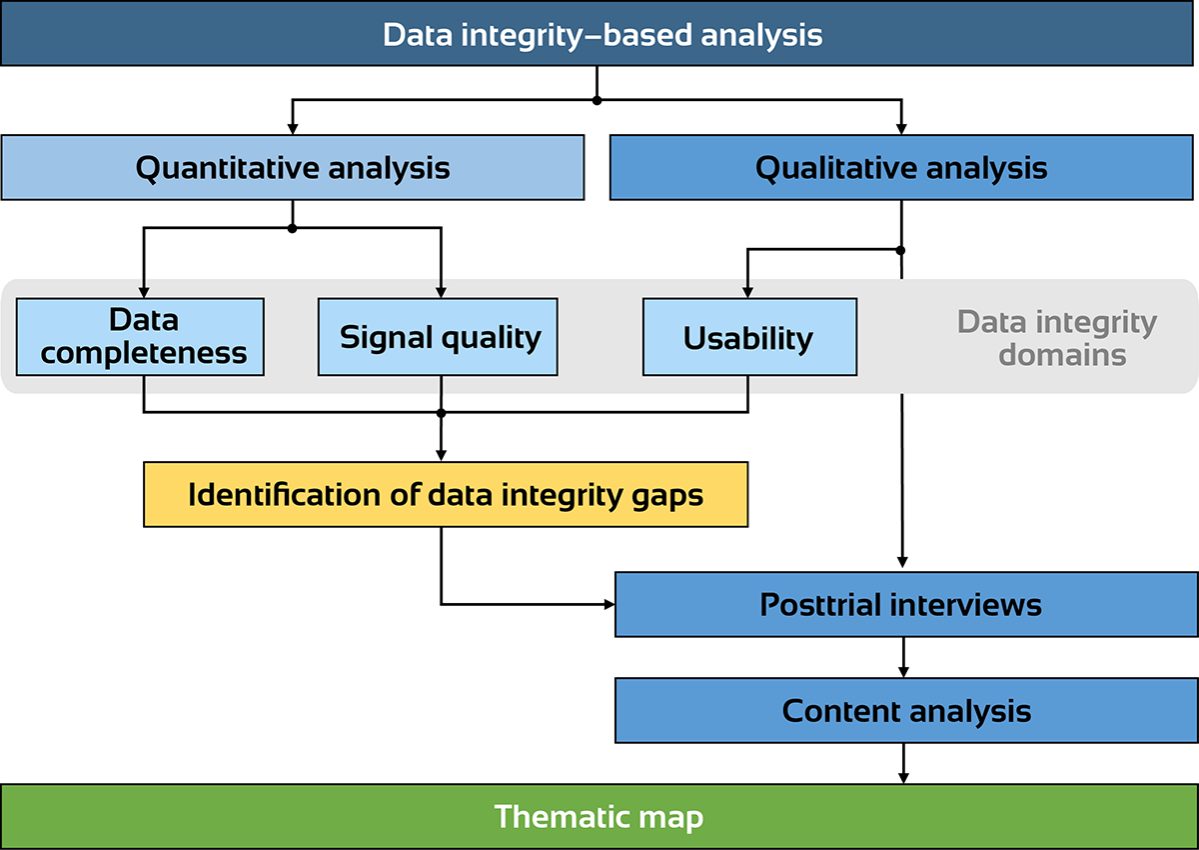
The flowchart demonstrates the data integrity–based analysis for identifying the source of data integrity loss.

To identify sources of data integrity loss, we retrospectively analyzed physiological data collected from a randomized controlled trial that implemented sensor-based mHealth tools to assess health outcomes in a rural setting [21]. After the analysis of the data integrity gaps in the recorded data, we identified possible causes that could have led to these gaps from both the paper-based trial case report forms (CRFs) and through qualitative data obtained from posttrial semistructured interviews with the app users conducted on site after the trial. The method development process is shown in Figure 1.

### Data Collection

We retrospectively analyzed physiological data and paper-based CRFs collected during a randomized controlled trial conducted in 82 Peruvian rural communities [21]. The trial was approved by the Universidad Peruana Cayetano Heredia ethical review board and the Cajamarca Regional Health Authority. The trial was registered on the ISRCTN registry (ISRCTN26548981).

A total of 317 children aged between 6 and 36 months were enrolled, and informed written consent was obtained from the children’s guardians. A total of 9 field workers (FWs) were trained to visit the children on 7 fixed geographical routes. Children were preassigned to these routes and visited in parallel by FWs once a week during the course of 60 weeks (6 weeks pilot, followed by a 54-weeks trial from February 2016 to May 2017, excluding 4 weeks of public holidays). To reduce the possibility of a courtesy bias, the routes of the FWs were rotated every 2 months.

During the weekly visits, FWs filled out a CRF and recorded physiological measurements with an mHealth app developed with LambdaNative (University of British Columbia, Canada) [22]. The app was installed on a tablet (Lenovo TAB 2 A7-10, Lenovo, CN) and connected to an external pulse oximetry sensor (iSpO_2_ Rx, Masimo International, Neuchatel, CH). FWs placed the multisite Y probe on the child’s thumb, index finger, or sole of a foot for the measurement of photoplethymogram (PPG), HR, and SpO_2_. The FW also measured RR with the same app by tapping on the touch sensitive screen of the tablet with each inhalation phase of breathing while observing the child’s bared belly [6]. All data collection procedures and interactions with the guardians and the child were subject of the informed consent and were approved by the ethics board.

The global positioning system sensor of the tablet registered the location where the visits took place (usually at the subject’s home). The assigned identification codes for children and FWs, date, and time were recorded with the app and the CRF. Furthermore, the health status of the child in the preceding week (maximum 2-week recall), the availability of the child (eg, absent from home), and unexpected sensor- or app-related technical problems during the visit were annotated in the CRF. Field coordinators conversed daily with FWs whether any problems occurred during the day, downloaded data, tested the sensors, and charged the tablets for the following day.

In addition to the assessments by the FWs, health personnel from 22 health care centers in the trial’s catchment area used the same tablets and software to collect physiological data in their consultations. The FWs received a 5-day initial training for tablet and CRF data collection with monthly retraining sessions of 2 hours. The health care center personnel were initially trained in 2 group sessions. Due to frequent changes of personnel in health care centers, new staff was retrained individually on site and physiological data were downloaded on a monthly basis.

### Quantitative Data Analysis

We quantitatively evaluated data completeness and signal quality of the physiological data and CRFs completed by FWs (N=9) with Matlab (R2016b, MathWorks Inc, Natick, Massachusetts, USA).

#### Data Completeness

We analyzed the completeness of home visit data and explored reasons for missing data. For this assessment, we considered a child no longer contributing to our data integrity analysis if there were no visits available for more than 8 consecutive weeks during the main trial period. We analyzed the tablet and CRF data separately. We considered the visit as missed if there were no tablet or CRF entries during a given week. We compared the data completeness between the pilot trial and main trial to assess training effects and improvements because of feedback from the pilot period. In the case of missing visits, we consulted with the field coordinator that was responsible for the FWs route planning for possible reasons. In addition, we reviewed the CRFs for potential explanations for the missing visits or recordings. For health care center recordings, we investigated barriers of using the tablet from interviews with the staff members.

#### Signal Quality

We evaluated the signal quality of the waveform obtained from the pulse oximeter. We calculated a signal quality index (SQI) using the established cross-validation based on morphological features and short-term variations [23]. We classified the PPG signal into 2 quality categories. We defined PPG signals that had sufficient quality to extract SpO_2_ values as “sufficient” (time series with high SQI for consecutive 8 seconds or longer) and the remaining as “insufficient”. To evaluate the performance across FWs over time, we evaluated the PPG signal quality for each FW separately. We calculated a “sufficient” PPG ratio over the total number of PPG signals within a sliding window of 40 recordings and a step size of 8 recordings. We chose these specific numbers because ideally each of the FWs should have obtained approximately 40 recordings per week and 8 recordings per day.

### Qualitative Data Analysis

We conducted semistructured posttrial interviews with the 7 FWs who were last to complete the children’s visits to assess their routines, experiences, and problems encountered during data collection. In addition, we conducted interviews with 7 health care center staff members (nurses or technicians) who were trained to use the tablet and worked at 7 different health care centers. These 7 health care centers were selected because of their varied geographical distribution, infrastructure, and load of patients. We assessed the frequencies and difficulties of using the tablet (see Multimedia Appendix 1). JZ and MM conducted the face-to-face interviews. Questions were asked in English and translated into Spanish during the interviews. All interviews were recorded with written notes and later digitalized by JZ and MM. Spontaneous follow-up questions and answers were also included in the analysis. Furthermore, we investigated potential usability issues that were not identified during the app development and trial pilot phase as well as whether the users had any difficulties using the tablet.

We conducted a content analysis [24] on the qualitative data, resulting in predominant themes around potential reasons that could affect the 3 main sources for data integrity (data completeness, signal quality, and usability). JZ collected and familiarized with the data, coded the reasons, and searched for themes. The final themes were discussed with LT and WK and reviewed by DM and WK. JZ then created a thematic map of potential reasons that could cause insufficient data integrity by identifying commonalities among all codes.

### Generalizing Risk Factors and Checklist Development

We systematically evaluated the obtained challenge categories to derive a methodology that could guide the pre-implementation evaluation of risks for general physiological sensing projects. We interpreted the main themes generated from the thematic map as challenges to be assessed and detailed each of them into specific risk factors based on the observed experiences during the trial. The risk factors were aggregated by JZ into a checklist draft.

To generalize the risk factors in this pulse oximetry–based checklist draft to other physiological sensing approaches, we selected 8 studies [25-32] that we considered representative of medical sensors–based mHealth projects (details are listed in Multimedia Appendix 2). A total of 4 graduate students (JB, SH, MM, and NN) with experience in conducting projects in low-resource settings reviewed and evaluated 2 selected projects each and applied the checklist to the selected projects. The list of risk factors was expanded with factors that were missing, either identified by the authors of the reviewed projects or from the reviewers’ own experiences. The wording and usability issues of the checklist were improved based on the feedback from the reviewers.

A total of 5 researchers (AA, DC, KK, WK, and BP) with proven practical experience in global mHealth implementation were invited by email to join an expert panel, assess the checklist, and provide feedback in 2 evaluation rounds. The first round was conducted via email to collect individual feedback on the checklist and suggestions for change from each expert. JZ aggregated all feedback into a point-by-point list of change recommendations and distributed it to all experts for review before the second round. The second round consisted of a group discussion that was conducted via videoconference. The list was presented point-by-point to the experts (JZ) and in case of disagreements between experts, discussed until a final agreement was reached. JZ translated decisions on changes into the checklist, which was then distributed to experts for final approval.

## Results

### Quantitative Data Analysis

**Figure 2:**
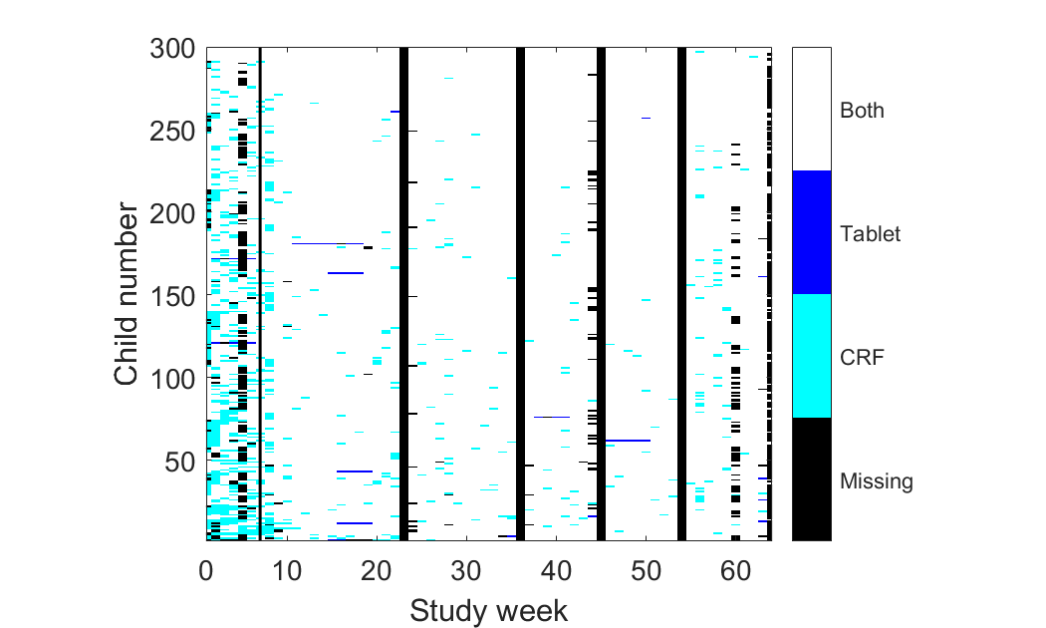
Visits obtained from tablet and case report form (CRF) entries over study weeks. The pixels in the order of legend sequence denote Both: visits registered both with the tablet and in the case report form, Tablet: visits only entered in the tablet, CRF: visits only entered in the CRF, and Missing: no visit recorded with either tablet or CRF. The continuous black lines indicate 4 full weeks of public holidays in the trial region. Missing visits at week 60 were because of Easter vacation.

Data from 300 out of the 317 recruited children met the inclusion criteria for the quantitative data analysis. A total of 15,757 home visits were made to these children during the trial and 1589 during the pilot period (Figure 2). We observed a higher percentage of visits entered through CRFs during the trial (15,322/15,757, 97.27%) compared with the pilot (13,910/15,757, 88.28%; Table 1). FWs encountered the children at home in 13,802 (13,802/15,757, 87.59%) cases. In 1953 cases (1953/15,757, 12.39%), children were absent from home, and hence, no data could be collected.

**Table 1:** Overview of the quantitative data from the 300 included children with respect to data completeness and photoplethymogram signal quality collected in the field during the pilot and trial.

Visits		Pilot (N=1800^a^), n (%)	Trial (N=16,200^a^), n (%)
**Actual visits (tablet)**		**n=1182 (65.67)**	**n=15,439 (95.30)**
	Total PPG^b^	977 (82.66)	12,098 (78.36)
	Sufficient quality PPG	368 (37.67)	7653 (63.26)
	Insufficient quality PPG	609 (62.33)	4445 (36.74)
**Actual visits (CRF^c^)**		**n=1589 (88.28)**	**n=15,757 (97.27)**
	Successful visits	1212 (76.27)	13,802 (87.59)
	Unsuccessful visits	377 (23.73)	1953 (12.39)
	Unlabeled visits	0 (0.00)	2 (0.01)

^a^N values based on scheduled visits.

^b^PPG: photoplethymogram.

^c^CRF: case report form.

**Figure 3:**
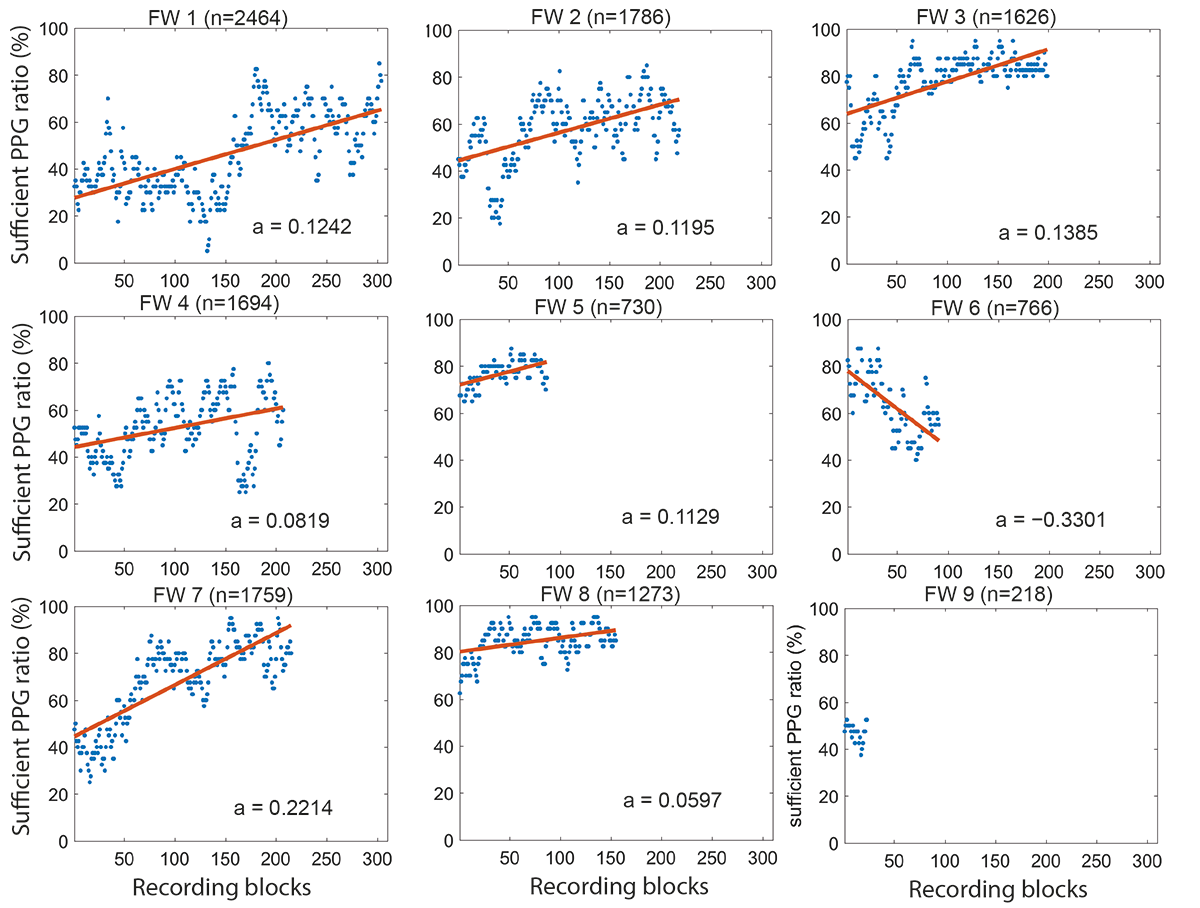
“Sufficient” photoplethymogram quality ratio over recording blocks for all 9 field workers (field worker 1-9 [n=number of photoplethymogram recordings performed]) during the trial. The blue dots depict the ratios between number of “sufficient” photoplethymogram signals and total number of photoplethymogram signals within each recording block (40 consecutive recordings with a step size of 8) by each field worker and the red trend lines are the linear fit of the ratios estimating the trend of recording quality (a=slope of trend line). Field worker 9 did not produce sufficient recordings for meaningful trend estimation and was not included in the signal quality analysis. FW: field worker; PPG: photoplethymogram.

Overall, 2 FWs left the study team during the trial. FW 5 left because of personal reasons after 6 months and was replaced by FW 6. FW 9 left already after 218 recordings that were insufficient for estimating a signal quality trend and, consequently, was excluded from the comparison. In total, the remaining 8 FWs recorded 82.66% (977/1182) PPG measurements during the pilot and 78.36% (12,098/15,439) PPG measurements during the trial (Table 1). For the trial, we classified 7653 (7653/12,098, 63.26%) PPG signals as “sufficient” and 4445 (4445/12,098, 36.74%) as “insufficient”. Of the 8 FWs, 7 increased their “sufficient” PPG ratio over time with a mean slope of 0.1226 (SD 0.0512; Figure 3).

### Qualitative Analysis

After the interviews with 7 FWs and 7 health care center staff members, we identified sources of low data integrity in 3 data integrity domains: (1) reasons regarding incomplete data, (2) low signal quality, and (3) usability issues.

#### Data Completeness

FWs encountered difficulties to find the correct routes to the family homes at the beginning of the pilot because of long distances and rough roads. To arrange efficient routes for each FW, the field coordinators evaluated the number of children per route, the actual duration to complete each route, and a rotation of FWs to share extra workload for routes to remote communities or hardship during difficult weather conditions. The pilot enabled to adjust the routes and refine data collection tools and protocols. After adaptation, we observed that a higher percentage of children were visited during the trial compared with the pilot. Furthermore, FWs noticed that if children were absent from their homes during the scheduled visits, it was mainly because the guardians had taken them to the fields as most of them were farmers.

FWs reported issues with the tablets and sensors, specifically the freezing of the app during measurements (3 FWs), that no pulse oximeter connection could be established (3 FWs) or unexpected insufficient tablet battery levels (1 FW) to perform all measurements as planned. All these issues were addressed by reporting to and solved daily by research assistants when FWs returned to the research station.

Another factor that hindered the measurement was guardians’ concern and preference not to let FWs interact with the children when the children resisted cooperating, when they were sick, or were sleeping. According to the CRFs, mothers did not allow measurements of the child in 305 cases. FWs also reported that when a child was sick, the mothers did not allow baring the child’s chest and abdomen to measure respiratory movement.

Most FWs (5 out of 7) perceived the lack of rapport with the child as a hindering factor at the beginning of the trial and after route rotations. They reported that the child was agitated and nervous and, therefore, resistant to interact. This problem was eventually solved and the trust between children and FWs built up over time.

In general, health care center staff were eager to use the tablet to measure the 3 parameters (HR, SpO_2_, and RR) using a single system. However, staff changes and extra workload were reasons for the low usage of the tablet. In 4 out of 7 health care centers where the interviews took place, the trained health care center staff member quit their job with the health service provider unexpectedly before a new staff member could be instructed to use the tablet. In addition, 1 health care center staff member indicated that health care center staff members were unable to spend extra time to collect measurements with the tablet because they had to complete their routine paper registrations and measurements for visiting patients with their regular medical devices.

#### Signal Quality

The FWs reported that cold fingers and movements of the children led to poor signal quality. For most of the visits when ambient temperatures were low, the pulse oximeter was not able to acquire a signal and the app indicated insufficient perfusion. With the progression of the study, FWs addressed this problem by warming the child’s finger before the measurement. The FWs also indicated that children tended to move after 10 seconds of measurements, leading to movement artifacts. In addition, children became nervous after approximately 3 unsuccessful measurement attempts and became less compliant.

#### Usability

Usability was primarily assessed in the pilot phase where the app was iteratively improved day-by-day in close interaction with the FWs. Workflow issues were addressed and data entry speed optimized. Translations of instructions from English to Spanish were confusing and, consequently, simplified.

A single FW reported that the font size of the selection list for demographic information (eg, the child’s communities and child’s identifier) was too small and the selection lists were too long to go through. The remaining FWs considered the app easy to use with a logical workflow.

### Thematic Map

**Figure 4:**
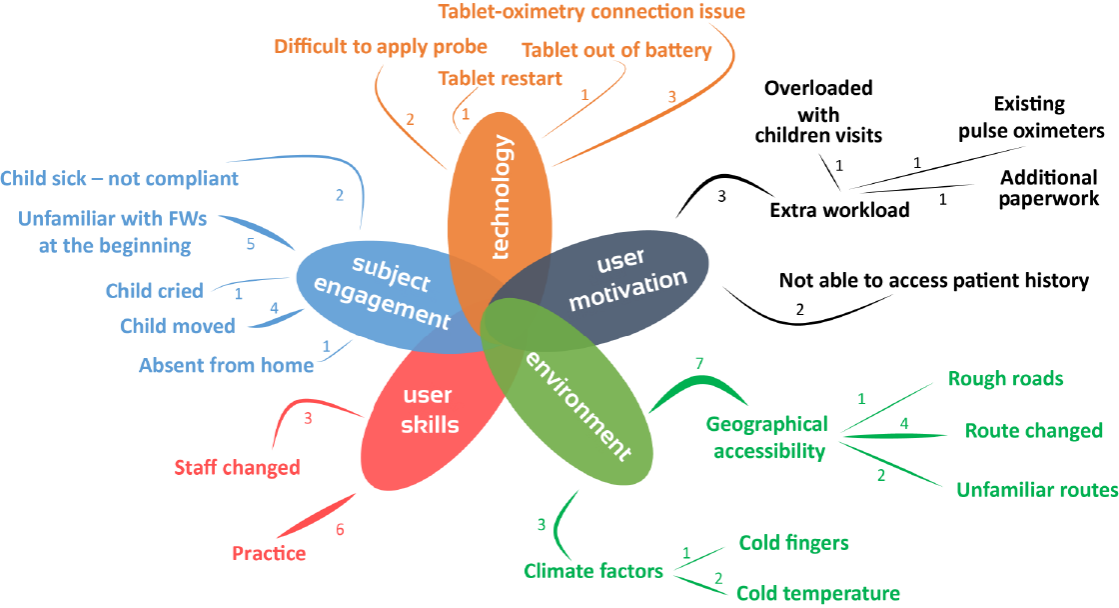
Thematic map showing 5 main challenge categories (technology, environment, user skills, user motivation, and subject engagement) generated by the related codes. The numbers along the connection lines indicate the frequency of occurrence of the codes, therefore, indicating the weight each code contributes to the main challenge. FW: field worker.

From the coded reasons for loss of data integrity in the 3 studied data integrity domains (data completeness, signal quality, and usability), we obtained 5 clusters: (1) environment, (2) technology, (3) user skills, (4) user motivation, and (5) subject engagement, which were represented in a thematic map (Figure 4). The strength of connections between codes denotes the frequency of occurrence of the codes and, therefore, illustrates the importance of a code within the cluster. We identified these 5 clusters as main challenge categories for the implementation of mHealth physiological monitoring in low-resource settings.

### Generalized Risk Factors and Evaluation Tool

The risk factors generated from the above-mentioned 5 challenge categories were expanded through further mHealth projects evaluation and expert reviews and were consolidated into a checklist. The checklist was divided into 5 sections that relate to the main challenge categories obtained from the thematic map and serves as an actionable evaluation tool. “Technology” considers technical aspects of the system, mobile devices, measurement devices or medical sensors, data management, software, and technical support. “Environment” takes into consideration the risks from climate, geography, culture, and society that can influence the quality of data collection and technology performance. “User skills” considers literacy, training, feedback, and retraining of the users. “User motivation” considers user availability and monitoring strategies to encourage user performance. “Subject engagement” focuses on the availability of the subject to be measured. Each section of the questionnaire features questions that can be answered with either a “yes,” “no,” “in progress,” or “not applicable (N/A).” By inspecting the “no” column of the checklist, the open issues and sources for potential risks can be visually identified. The checklist is available under a CreativeCommons NonCommercial ShareAlike licence as a printable PDF and an interactive Web-based form [32].

## Discussion

### Principal Findings

In this study, we evaluated implementation challenges of physiological monitoring with mobile sensors in low-resource settings and developed a data integrity–based methodology to evaluate the challenges according to the factors environment, technology, user skills, user motivation, and subject engagement. This methodology could, that is, in the form of the developed checklist, assist mHealth implementers to identify risks.

Until now, methodologies for systematically assessing implementation challenges in physiological monitoring enabled by mHealth did not exist. Implementation challenges were reported only intermittently covering training [10], limited resources [34], motivational barriers [35], language and cultural barriers, weak health systems, and limited external financing schemes [36]. With our approach that focuses on the exploration of challenges based on data integrity, we provide central themes that implementers can systematically follow. By exploring the causality of data integrity loss, the methodology provides a broad coverage of risks.

Environment- and technology-related challenges are closely linked and should be evaluated with respect to the following aspects: weather, geography, population, and related difficulties that influence the access to subjects as well as the mHealth tool’s functionality. Unlike text or voice message–based mHealth projects, where the mobile communication infrastructure is the major bottleneck that influences study outcomes [36], environmentally induced barriers such as missing subject recordings because of inconvenient transportation have large impact on sensor-based mHealth projects. Those factors should be carefully considered and potential solutions tested and planned for.

In addition, implementers should plan for sufficient follow-up and technical support during the lifetime of a project. In our case, the cold climate made the children feel uncomfortable to bare their chest and abdomen and, in addition, cold fingers negatively influenced the signal strength. This problem could be addressed by considering whether the chosen sensing modalities are suitable for the local settings. Moreover, from our experience, good preparation includes collaborating and exchanging information with all stakeholders (parents, caregivers, and health care center personnel) early in the process, which helps to evaluate the feasibility of the chosen system and methods before implementation [27].

To solve user skill–related challenges, sufficient training of the users, understanding their opinions and attitudes toward devices and systems, as well as assisting them in fostering a good relationship with the subjects are essential. The “sufficient” PPG ratio for all except 1 FW increased across the study period, indicating a positive correlation between the users’ experience level and achieved signal quality. FW 6 who had a negative trend in signal quality was hired midtrial and was not part of the extensive training during the pilot study. Therefore, we cannot exclude the fact that the training provided at the appointment was insufficient. The posttrial interview with FW 6 did not reveal a clear reason why the decreasing trend could have happened. Therefore, further investigations will be needed. This was the first time that mHealth technology was introduced into the trial region. Although mobile phones were widely used in this area, the sensor-related mHealth tools were new to the users (FWs). We recommend training the users to apply the sensors within the target environment to ensure they are fully comfortable with the functionality and able to perform minor troubleshooting themselves as well as perform regular refresher trainings. In addition, implementers should develop evaluation methods to track and supervise the performance of the users during the project’s lifetime and be prepared to receive feedback from users. This way, users can be trained and retrained based on specific issues encountered with the aim of increasing data quality and efficiency.

The subject engagement challenges relate to the level of cooperation between users and subjects. The positive engagement is one of the most important factors that contribute to data completeness. Moreover, medical sensors are sensitive to motion artifacts; therefore, collecting measurements from pediatric populations highly depends on their willingness to cooperate. First of all, the user should establish a good relationship with the subjects. We preemptively considered this as an important factor and conducted extensive pretrial training for FWs in 2 kindergartens and day cares to familiarize them with working with children. Our FWs tried to establish a friendly rapport and played games with the children to calm them before measurements. In general, users should practice measurements on the targeted subject population to optimally perform measurements while creating a conducive environment. In addition, communication with and gaining support from subjects’ family members are essential. In our case, the parents’ support in general was high. In this trial, no cultural groups rejected participation. For pediatric studies, parents should be encouraged to support the mHealth users in handling their children.

Although health care centers in low-resource settings are eager to use technological support to assist clinical measurements, the users faced motivational challenges. On the one hand, supervised training and observable benefits for the staff might increase their motivation to use the new technology. Haberer et al show that sufficient training and improved skills increase the motivation of users [37]. On the other hand, strong motivation also increases lay workers’ performance. Mwendwa et al suggest that poor performance of the community health care workers cannot be solely solved by training skills but also by highlighting the consequences of the measurements and explaining the process of data collection [38]. A properly supervised training and explanations of the benefits of the mHealth tool have the potential to increase user motivation. However, as Graham et al identified in their recent study on implementation of handheld pulse oximetry in Nigerian hospitals, provision of equipment and training alone is not enough [39]. Reminders and encouragement of peers are needed as increased workload burden and technical difficulties were negatively influencing motivation to adopt pulse oximetry. Although these findings were not obtained from an mHealth implementation study, we have good reason to believe that this applies to technology implementation in general, including mHealth.

Checklists have been proven to raise awareness and prevent incidents of certain reoccurring issues. Pilots and aircrew perform preflight checklists to improve flight safety [40]. The World Health Organization suggests using a surgical safety checklist in operating room environments to reduce the number of surgical incidents and deaths [40]. Other health care–related checklists were developed such as assessing the scalability of pilot projects [41], reporting health interventions [42], checking mHealth solutions [43], and monitoring and evaluating outcomes of digital interventions [44]. However, the effectiveness of a checklist depends on the complete implementation of recommended actions. Van Klei et al showed that after applying the surgical safety checklist in operating rooms, the mortality rate only reduced significantly for those surgeons who fully completed the checklist [45]. Furthermore, to effectively distribute the checklist to targeted audiences as well as encourage its use is challenging. Therefore, we provide a tool online for easy and efficient assessment.

Historically, widespread adoption of mHealth tools is limited with too many proof-of-concept projects not achieving sustainable implementations and often lacking evidence to justify scaling [2]. The main challenge categories covered by our methodology coincide with the critical factors for success in scaling medical mobile technologies identified by Lundin and Dumont [46]. Besides understanding the needs from the local area, integrating the technology into the local health care systems, engaging end users, and involving all related stakeholders, other factors that are not driven by data integrity (eg, finance-related factors) can determine the scaling success of mHealth projects.

### Limitations

Our methodology development is based on the physiological measurements performed in a single trial limited to pulse oximetry and RR measurements. Therefore, the 5 identified sources for loss of data integrity may not be equally weighted in other projects. For example, in a user self-management project, where the mHealth user is also the studied subject, the aspects of training and education become more important and, therefore, might require a stronger emphasis. Furthermore, although the monitored trial implemented mHealth tools, it did not aim at scaling the usage of the tools. A scaling project could have, because of its extension to multiple geographical locations spanning over different health districts, slightly different aims and would have more sophisticated monitoring tools in place. Our methodology might have not comprehensively captured these aims. However, as our methodology is based on data integrity, the evaluation approach can be easily expanded to these differences.

We were not able to validate the effectiveness of the provided checklist prospectively on a large number of projects. We tested and expanded the checklist extensively by reviewing multiple published projects implementing medical sensors by using early drafts of the checklist and complementing missing aspects. Furthermore, an invited panel of experts evaluated and complemented the checklist with missing aspects based on their own diverse expertise. To promote adoption and collect feedback from early adopters, we have published the checklist online.

### Outlook

To enable a dynamic growth of the checklist, we provide a digital form of the checklist online where anonymous usage of the checklist is tracked. We plan to use these data, together with direct feedback from implementers, to improve the checklist in regular intervals and redistribute updated versions through the same platform. As there is currently a lack of target product profiles for sensor-based mHealth systems in many disease management apps and our checklist is developed for implementers to reduce the risk of data integrity loss, we would like to explore the potential of the checklist to serve as a reference for building target product profiles that call for high degrees of data integrity.

### Conclusions

Introducing physiological monitoring with mHealth tools into low-resource settings can deliver simple and effective sensing technologies to improve objectivity of health assessments but faces challenges on multiple levels. The target environment, appropriateness of the technology, the skills and motivation of the user, as well as the subject engagements influence the implementation of mHealth solutions alike. With our newly developed methodology and its derived checklist, we enable project implementers to follow a structured evaluation protocol, identify potential risks, and reevaluate challenges during implementation. Such a systematic evaluation of challenges could also be applied and adapted to other areas in the rapidly growing digital health field.

## Appendix

Multimedia Appendix 1 Semistructured interview questions.

Multimedia Appendix 2 Overview of the mobile health (mHealth) projects used for testing and reviewing the checklist.

## Abbreviations

CRF: case report form
FW: field worker
HR: heart rate
mHealth: mobile health
PPG: photoplethymogram
RR: respiratory rate
SpO_2_: peripheral capillary oxygen saturation
SQI: signal quality index
