# Supplementary material for: Data Integrity–Based Methodology and Checklist for Identifying Implementation Risks of Physiological Sensing in Mobile Health Projects: Quantitative and Qualitative Analysis
Source: JMIR Mhealth Uhealth. 2018 Dec 14;6(12):e11896. doi: 10.2196/11896 (PMC6315242; doi:10.2196/11896)
Supplement: Multimedia Appendix 1 [file mhealth_v6i12e11896_app1.pdf]

Appendix I: semi-structured post-trial interview questions.

a. Tablet-based data collection in rural Andean Peru: semi-structured post-trial interview with 7 fieldworkers on technical and inter-personal experiences with the household-level application of an mHealth data collection device.

1. Can you tell you us about the procedure of visiting the children?
2. Why do you have missing entries on the tablet?
3. Did you have any problems in the beginning of the study?
4. Do you have any difficulty in using the tablet?
5. Is there any problem with the measurement?
6. What were opinions from the families?
7. Do you have other general suggestions about the study?

b. Semi-structured post-trial interview questions asked to trained medical staffs at the 7 health care centers, San Marcos, Peru.

1. How often do you use the tablet?
2. How many kids do you measure with the tablet per day?
3. How many kids visit the health care center per day?
4. Tell us your routine of doing measurements with the tablet?
5. Can you perform an example measurement (on me or on a kid if there is one)?
6. Do you have any difficulty in using the tablet?
7. Do you have any suggestions for improvement?
