# Supplementary material for: Data Integrity–Based Methodology and Checklist for Identifying Implementation Risks of Physiological Sensing in Mobile Health Projects: Quantitative and Qualitative Analysis
Source: JMIR Mhealth Uhealth. 2018 Dec 14;6(12):e11896. doi: 10.2196/11896 (PMC6315242; doi:10.2196/11896)
Supplement: Multimedia Appendix 2 [file mhealth_v6i12e11896_app2.pdf]

Appendix II: Overview of the mHealth projects used for testing and reviewing the checklist.

Ref	Title	Year	Country	Parameters and/or mobile sensors	Evaluator
[25]	Implementation of a Real-Time Human Movement Classifier Using a Triaxial Accelerometer for Ambulatory Monitoring	2006	Australia	Human movement, waist-mounted triaxial accelerometer unit	MM
[26]	DialBetics: A Novel Smartphone-based Self-management Support System for Type 2 Diabetes Patients	2014	Japan	Blood pressure, body weight, blood glucose, pedometer counts, photos	MM
[27]	Building quality mHealth for low resource settings	2016	South Africa	Mobile app based clinical guidelines	SH
[28]	Development of mHealth Applications for Pre-Eclampsia Triage	2014	South Africa	Phone oximeter	SH
[32]	Development and Internal Validation of a Predictive Model Including Pulse Oximetry for Hospitalization of Under-Five Children in Bangladesh	2015	Bangladesh	Pulse oximeter	NN
[29]	Usability and Feasibility of PIERS on the Move: An mHealth App for Pre-Eclampsia Triage	2015	South Africa	Mobile app with integrated pulse oximeter	NN
[30]	3 Development and testing of pictograms for the symptoms of pre-eclampsia in Ogun State, Nigeria	2016	Nigeria	Pictograms representing symptoms of pre-eclampsia	JB
[31]	Mobile Phone Text Messages to Support Treatment Adherence in Adults With High Blood Pressure (SMS-Text Adherence Support [StAR]) A Single-Blind, Randomized Trial	2016	South Africa	Blood pressure;<br>Mobile phone text messages	JB
